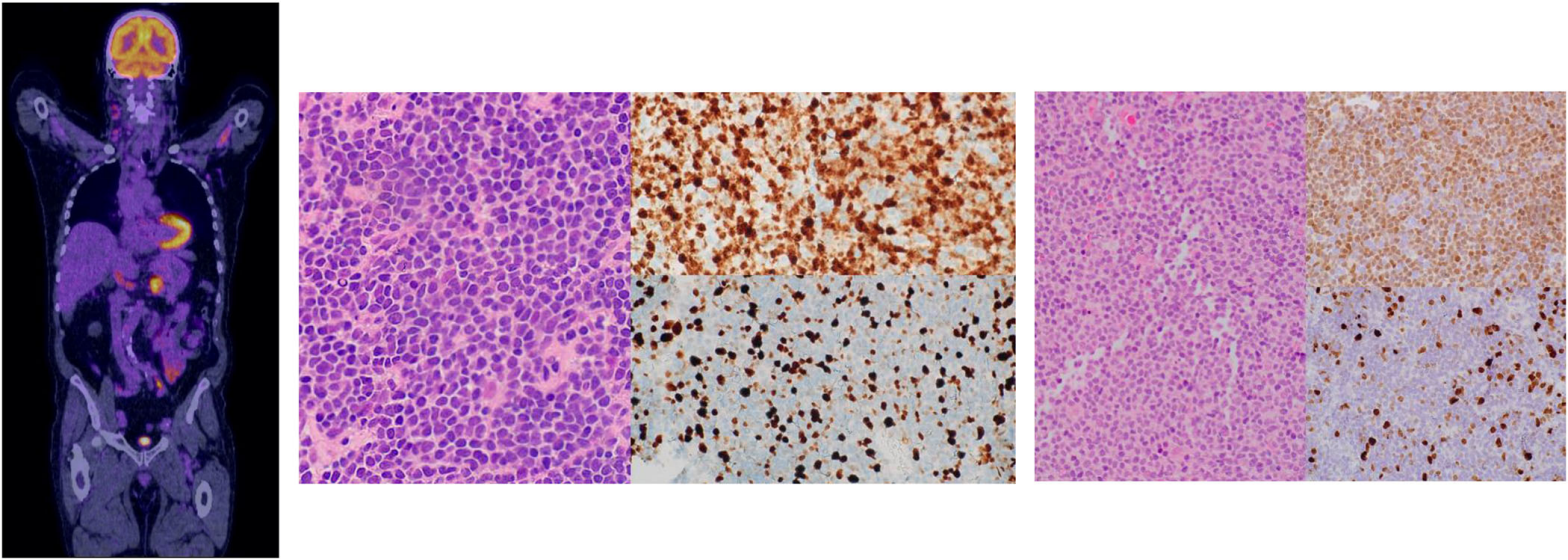# Discordance between positron emission tomography standard uptake value and proliferation index in mantle cell lymphoma: An initial communication

**DOI:** 10.1002/jha2.348

**Published:** 2021-11-28

**Authors:** Alexandra Renn, Andrew Wotherspoon, Ayoma D. Attygalle, Katherine Vroobel, David Cunningham, Bhupinder Sharma

**Affiliations:** ^1^ The Royal Marsden Hospital London UK; ^2^ The Institute of Cancer Research London UK

An 80‐year‐old male complained of rapidly enlarging neck lymphadenopathy. Following ultrasound‐guided biopsy, mantle cell lymphoma (MCL) was diagnosed. Staging fluorodeoxyglucose (FDG)‐positron emission tomography (PET) demonstrated avid supra‐ and infra‐diaphragmatic adenopathy. However, PET showed striking heterogeneity of standard uptake value maximum (SUV_max_; Figure 1, left display): the biopsied cervical node (28 mm) had a moderate SUV_max_ of 5.5, whereas a peripancreatic node (35 mm) had an intense SUV_max_ of 11.5. A *watchful waiting* strategy had been advocated based on the low proliferation index of the biopsied neck node. However, the high SUV_max_ of the abdominal node raised concern for high proliferation index MCL (warranting therapy), therefore additional abdominal biopsy was undertaken. Histological analysis of both the neck (Figure 1, middle display) and abdominal node (Figure 1, right display) confirmed concordant low proliferation index disease, demonstrating MCL classic morphology (haematoxylin and eosin stained section, 400× objective; Figure 1, left panel) that is cyclin D1 positive (Figure 1, right upper panel; 400× objective) with a low proliferation index of 15% (400× objective).

MCL is a distinct B‐cell non‐Hodgkin lymphoma with a poor clinical outcome; the median survival is 3 years and definitive cure is rare. The genetic hallmark of MCL is the chromosomal translocation t(11;14) resulting in aberrant expression of cyclin D1. Assessment of the Ki‐67 index is critical, being of prognostic impact and associated with poor survival in MCL patients (a high Ki‐67 index is considered >30%).

The discordance of the imaging and histological findings on this early observation suggests that FDG‐PET may exhibit a weak correlation between SUV_max_ and Ki‐67 expression in patients with MCL; to our knowledge, data correlating SUV_max_ and Ki‐67 have not previously been published. An accurate imaging biomarker tool for proliferation index in MCL would be clinically useful; we support further research to assess whether there is a correlation between SUV_max_ and Ki‐67. We also posit the novel tool of diffusion weighted imaging with apparent diffusion coefficient measurement may have potential to assess proliferation index in MCL.

**FIGURE 1:**